# Preparation and antimicrobial activity of ZnO-NPs coated cotton/starch and their functionalized ZnO-Ag/cotton and Zn(II) curcumin/cotton materials

**DOI:** 10.1038/s41598-020-61306-6

**Published:** 2020-03-25

**Authors:** Issa M. El-Nahhal, Jamil Salem, Rawan Anbar, Fawzi S. Kodeh, Abedelraouf Elmanama

**Affiliations:** 1 Department of Chemistry, Al-Azhar University, P O Box 1277 Gaza, Palestine; 2 Department of Medical laboratory sciences, Islamic University of Gaza, P.O Box 108 Gaza, Palestine

**Keywords:** Preventive medicine, Biomedical materials, Structural properties

## Abstract

ZnO-NPs coated cotton or starched cotton fibers were successfully prepared via ultrasound irradiation. Different concentrations of soluble corn starch (1–3 starch wt.%) were used to stabilize ZnO-NPs onto the surface of cotton fabrics as entrapped species. The use of none-toxic biocompatible starch has improved the adhesion properties of the cotton fibers towards ZnO-NPs. This also enhanced the durability of ZnO-NPs onto the cotton fabrics and decreased their leaching from the surface of cotton fabrics. When 3 starch wt.% solution was used, deposition of ZnO-NP increased by 53% after 10 washing cycles. The antibacterial activity against Staphylococcus aureus and Escherichia coli increased by 50 and 21.5%, respectively. Functionalization of ZnO coated cotton with silver nanoparticles (Ag-NPs) and curcumin results in formation of ZnO-Ag/cotton and Zn(II) curcumin/cotton composites. The functionalized nanocomposites ZnO-Ag coated cotton material showed a synergistic antimicrobial behavior than that of individual ZnO/cotton material. The Zn(II) curcumin complex coated cotton showed higher antibacterial activities against both Staphylococcus aureus (Gram-positive) and Escherichia coli (Gram-negative) bacteria than that of the ZnO/cotton material.

## Introduction

The resistance of microbial activity against classical antimicrobial therapies increased due to increase in the ability of these organisms to develop resistance to virtually all antimicrobial systems^1^. New strategies are recently considered in order to combat bacteria resistance by using various types of inorganic metals or metal oxides coated textiles to impart their antimicrobial activity. The new materials showed high stability and antibacterial effectiveness even after intensive laundry regimes are employed in hospitals^2,3^. Recently some inorganic metal oxides^4–10^ and metals nanoparticles coated cotton composites have attracted attention, due to significant antimicrobial activities against pathogenic bacteria^11–14^. Alternative inorganic nanocomposite materials have gained interest due to their safety and selective toxicity against bacteria^10,15–19^. Special attention has been directed toward the use of antibacterial coated fabrics e.g. medical clothes to minimize the chance of nosocomial infections^20–22^. The main problem is to prepare stable metal oxide coated cotton composites, less leachable, and more effective for elimination of microbial pathogen. Natural biodegradable and biocompatible biopolymers chemically linked with metal oxides have been recently used to enhance the nanoparticles stability^23–25^. The use of the various agents e.g. enzyme, chemicals or binding agents as tools for activation of textiles may result in changes in the nature of the cotton fibers^26–28^. A single step sono-enzymatic process for coating cotton medical textiles with antibacterial ZnO nanoparticles (NPs) and garlic acid(GA) was recently described to produce biocompatible fabrics with durable antibacterial properties^2^. This system showed a better fixation for ZnO onto the cotton substrate and better antimicrobial activity^2^. Harsh chemicals and enzymes or binding agent *in-situ* synthesis of metal oxides coated cotton are not recommended. Instead of that, different surfactants that include: cationic anionic and non-anionic were used in coating process^3,29^. Surfactants were used to stabilize the MO-NPs by controlling their shape and size as encapsulated species. Among these methods, we have focused on the use of polymeric binder such as corn starch to accomplish two tasks simultaneously: enhance the adhesion properties of the cotton and immobilizing the ZnO-NPs.

**Figure 1:**
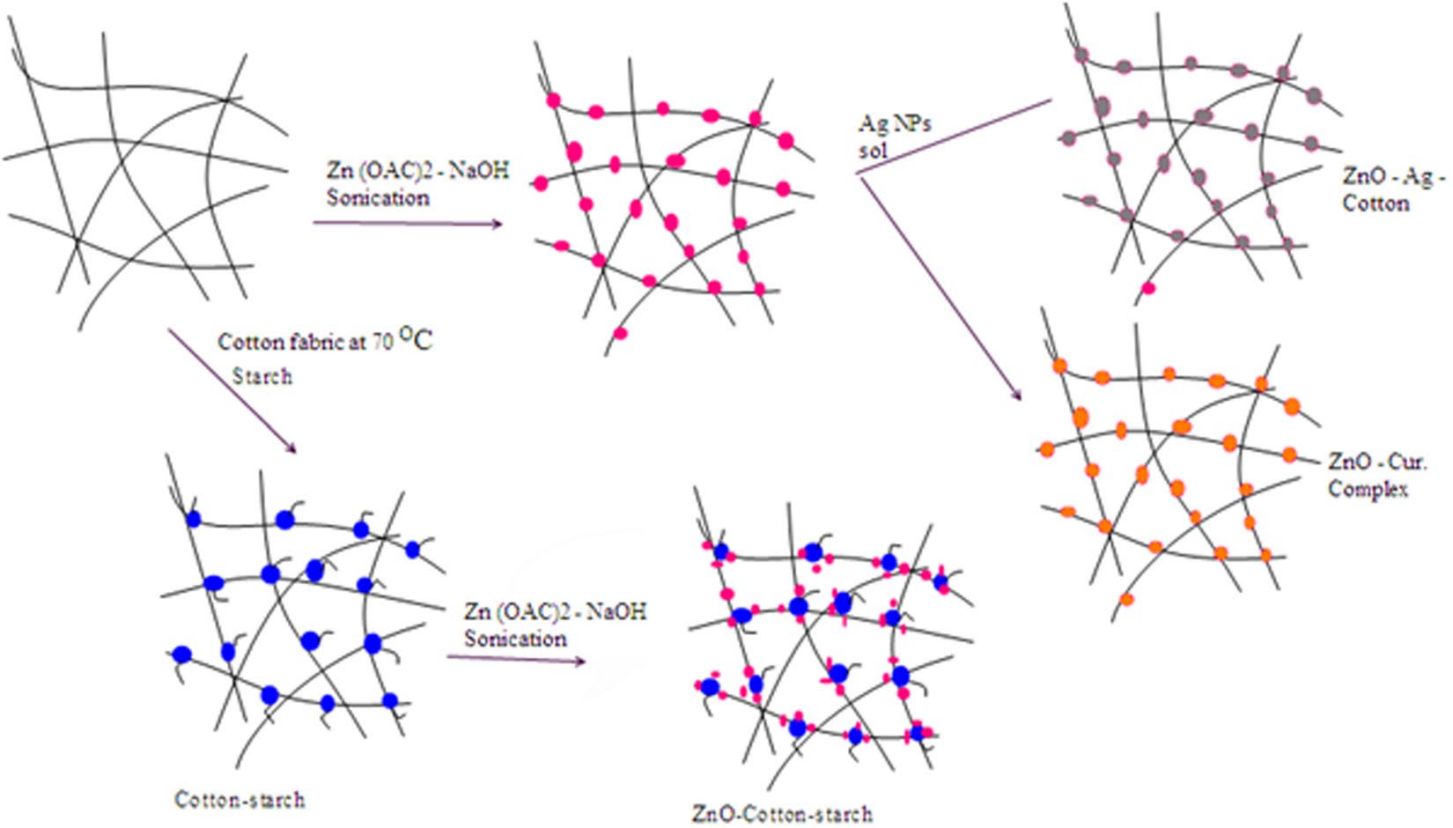
Schematic description of deposition of ZnO-NPs, ZnO-Ag nanocomposite and Zn(II) curcumin complex onto cotton fibers.

In this present research, corn starch (1–3 starch wt.%) as none toxic material was used to enhance adhesion properties of the cotton fiber surface^30^, for loading zinc oxide nanoparticles. The formation of ZnO-NPs coated cotton and starched cotton fibers and its functionality materials, ZnO-Ag and Zn(II) curcumin are described in Fig. 1. The durability results showed that starched cotton samples exhibit higher deposition of zinc oxide nanoparticles and higher antimicrobial activity than the corresponding samples prepared without using starch. Minimum leaching of zinc oxide from the fabrics was observed even after 10 washings cycles. In the second part of the experimental, the ZnO/cotton (natural cotton) was functionalized with Ag-NPs or curcumin^31–33^, where two new stable composites (ZnO-Ag/cotton and Zn(II) curcumin/cotton) were prepared (scheme 1) and their antimicrobial activity were examined. We have noted that none of Zn or Ag are leaked out from ZnO-Ag/cotton or Zn(II) curcumin coated cotton.

The morphology of the cotton coated materials and their chemical structure have been analyzed by SEM, TEM and XRD. Although common antimicrobials agents e.g. penicillin and tetracycline have been well known as antimicrobials, bacteria have now become resistant to most antimicrobial agents and hence the main future research in antimicrobial therapy is to develop novel materials which may work as effective antimicrobials. In this context, ZnO-coated cotton has been synthesized and further functionalized with silver nanoparticles and curcumin. The antimicrobial activity for the modified materials (ZnO-Ag and Zn(II) curcumin) was enhanced as compared with the parent ZnO/cotton. This is probably that ZnO-Ag illustrated synergistic behavior and enhanced antimicrobial activity for both gram-positive and gram- negative.

## Experimental and Methodology

### Chemicals and reagents

Zinc acetate di-hydrated (Zn(CH_3_COO)_2_.2H_2_O) and sodium hydroxide (NaOH) were purchased from HiMedia, India. Starch (from corn) was purchased from Merck. A Turkish cotton (100% cotton) was purchased from local market and pretreated before used. Dehydrated nutrient agar powder, nutrient broth powder, Sabouraud Dextrose Agar (SDA), Di-Chloran Rose Bengal Chloramphenicol (DRBC) agar and Potato Dextrose Agar (PDA), were purchased from (HiMedia, India) and used to prepare culture media.

### Analytical methods

Chemical analysis of the ZnO-NPs coated cotton and ZnO-NPs coated starched cotton and their functionalized materials (ZnO-Ag and Zn(II) curcumin complex) coated cotton were investigated using various techniques: scanning electron microscopy (SEM) using Carl Zeiss AG - EVO® 60), transmission electron microscope (TEM) JEM2010 (JEOL), X-ray diffraction (XRD) using EQuniox 3000, INEL, France and Ultraviolet-Visible (UV/VIS) Spectrophotometer using SHIMADZU-1601 model UV/VIS.

### Coating process

#### Preparation of starched cotton fibers

Cotton fibers were firstly purified by washing with 1% solution of sodium dodecyl sulfate (SDS) at 50 °C for 1 hr. The cotton fibers were then rinsed with distilled water several times, the fibers were dried under vacuum (0.1 torr) at 80 °C for 24 hrs. The dried cotton fibers were starched using three different concentrations (1–3 starch wt.%) by treating cotton fibers (1.0 g) with 20 mL of different concentrations (1–3 starch wt.%) of corn starch at 70 °C for 5 hrs. The starched cotton fibers were dried under vacuum (0.1torr) at 80 °C for 24 hrs.

#### Coated ZnO-NPs/cotton and ZnO-NPs/starched cotton materials

The ZnO-NPs coated cotton and ZnO-NPs coated starched cotton materials were prepared using previously described method^3,9,10^ by treating 0.50 g of cotton or starched cotton with zinc(II) acetate (0.05 mol) and NaOH (0.10 mol) in 30 mL deionized water^3,9,10^. The mixture was irradiated using Ultrasonicator (Model US-150 Ti-horn, 20 kHz, output 10 Turning 7) for 60 minutes with stirring from time to time to ensure that all cotton or starched cotton fabrics were homogeneously coated with ZnO-NPs. During sonication, flask was placed into a cooling bath keeping a constant temperature between (35–40 °C). ZnO-NPs coated cotton and ZnO-NPs coated starched cotton materials were then washed thoroughly several times with distilled water and dried at 80 °C overnight. These coated materials are labeled hereafter as ZnO/cotton and ZnO/starched cotton (1–3 starch wt.%).

#### Functionalized cotton and ZnO/cotton with curcumin

**Figure 2:**
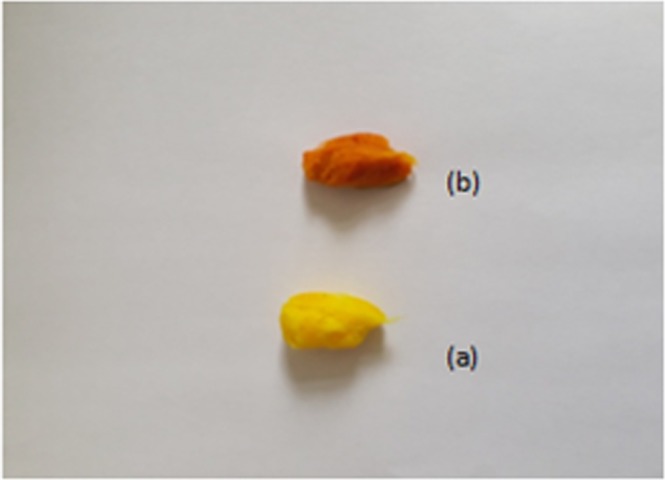
(**a**) Curcumin/cotton sample (**b**) Zn(II) curcumin complex/cotton sample.

200 mg of ZnO/cotton (10 washing cycles sample) was treated 10 ml of ethanol solution of curcumin (2.71*10^−3^ M). A yellow curcumin/cotton and orange zinc(II) curcumin complex coated cotton were formed (Fig. 2). The curcumin coated cotton and Zn(II) curcumin complex coated cotton were washed with three successive portions of 20 ml ethanol to remove free curcumin, then dried at 70 °C under vacuum (0.1torr) for 24 hrs.

#### Preparation of ZnO-Ag nanocomposite coated cotton

**Figure 3:**
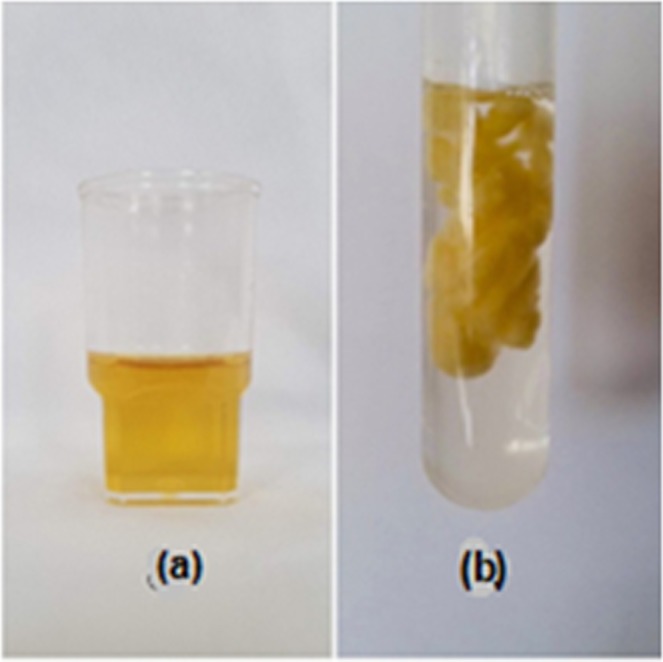
(**a**) Ag-NPs sol (**b**) ZnO-Ag/coated cotton sample.

ZnO-Ag nanocomposite coated cotton was prepared by treatment of 200 mg of ZnO-coated cotton (10 washing cycles) with 10 ml of Ag-NPs sol (yellow color)^31,32^. The mixture was irradiated using an Ultrasonicator for about 15 minutes to ensure coating with Ag-NPs (Fig. 3). The ZnO-Ag nanocomposite coated cotton material was washed with two successive portions of 20 ml distilled water to remove the unreacted Ag-NPs and dried at 70 °C under vacuum (0.1torr) for 24 hrs.

#### Wash durability tests

**Figure 4:**
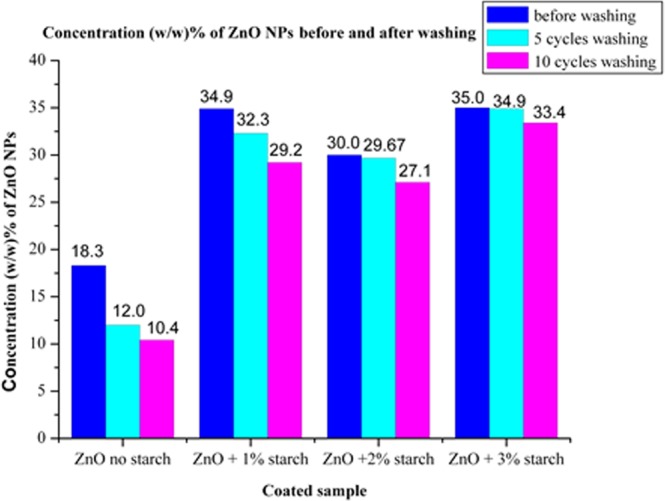
Content of ZnO-NPs (w%) before and after five and ten washing cycles.

Wash durability tests for ZnO/cotton and ZnO/starched cotton at three different starch concentration (1–3 starch wt%) were carried out using distilled water at ambient temperature (22 °C) (Fig. 4). The (w/w) % of ZnO-NPs before and after 5 & 10 washing cycles was determined using the kit method in a triplicate.

#### Antimicrobial activity tests

Zinc coated cotton fabrics were subjected to the assessment of their antibacterial activity before and after two washing cycles processes using similar methods previously described^3,29^. The antimicrobial activity of ZnO/cotton and ZnO/starched cotton was tested against the Gram-negative *Escherichia coli* and the Gram-positive *Staphylococcus aureus*. The clinical isolates were kindly provided by the Microbiology laboratory of Al-Shifa’ Hospital. The antimicrobial activity was tested according to the standard quantitative test (AATCC 100, 2004) with some modifications. A piece of the coated cotton was tested against a known concentration of bacterial suspension and the reduction in the viable cells was calculated. A dry piece of coated or uncoated cotton material (100 mg) was used for the antimicrobial test. The pieces were sterilized by autoclaving and by UV-sterilization before testing the antimicrobial activity. The cotton materials “Test and Control “were soaked in sterile normal saline and incubated for a standard time without shaking. A preliminary antibacterial test was carried out for ZnO/starched cotton of different concentrations of starch (1–3 starch wt.%), where the cotton materials were washed with water very well and then allowed to dryness.

Two cotton pieces “Test and Control “were inoculated with 500 µL of the bacterial suspension and then each piece was inserted in a vial containing 20 ml of sterile physiologic saline solution (NaCl 0.9%). The vials were tightly closed and allowed to vigorous shaking for about 1 min. and then incubated at 37 °C for 24 hr. After incubation, 1000 µL of each sample was taken and serially diluted with (0.9%) NaCl solution and 100 µL of each dilution was transferred onto nutrient agar plates. The plates were allowed to grow overnight at 37 °C and the viable bacteria were counted.

## Results and Discussion

### Preparation of starched cotton fabrics (1–3 starch wt.%)

The interfacial adhesion^30^ requires the use of coupling agents between phases to avoid the poor adhesion between cotton fibers and metal oxides nanoparticles, this work is proposed to prepare starched fibers^30^. The starched cotton materials were prepared by treatment of pristine cotton with corn starch at three different concentrations (1–3 starch wt.%) at 70 °C for 5 hours, vacuum filtered and dried to obtain gelatinized starched cotton fibers.

### Preparation of ZnO-NPs coated cotton fibers

ZnO-NPs coated cotton fibers or ZnO-NPs coated starched cotton fibers were obtained by deposition of ZnO-NPs onto cotton or starched cotton fibers using the ultrasound irradiation method of the zinc oxide according to the reaction illustrated in Scheme 1^3,10,29^. In presence of NaOH as basic media, the cellulose (Cell-OH) is changed into cellulosate anion (Cell-O^−^), which became very active creating more sites to be attached with the zinc ions. The presence of basic media also promotes the formation of ZnO-NPs from the decomposition of Zn(OH)_2_ by means of ultrasonication. This is probably involved: formation of Zn(OH)_2_, which is absorbed onto the surface of cotton or starched cotton, transformation of Zn(OH)_2_ to ZnO-NPs by means of irradiation energy and removal the excess of reactants by washing with distilled water (DW). The role of starch is to stabilize the ZnO-NPs by controlling their size and shape.

### Wash durability tests

Wash durability tests were examined for the content ZnO-NPs coated cotton fabrics and compared with the corresponding ZnO-coated starched cotton (1–3 starch wt.%). The results showed that the content of coated ZnO-NPs have significantly increased from 18.3% to 35% when cotton-starched was used (Fig. 4). There is a remarkable reduction of leaching ZnO-NPs nanoparticles to 5% when starched cotton was used in comparison with 56.8% in absence of starch. Of three different wt. starch percentages (1,2,3 starch wt.%), starch of 3 wt.% showed the most minimizing leaching of ZnO-NPs.

### Structural characterization

#### Scanning electron microscopic (SEM)

**Figure 5:**
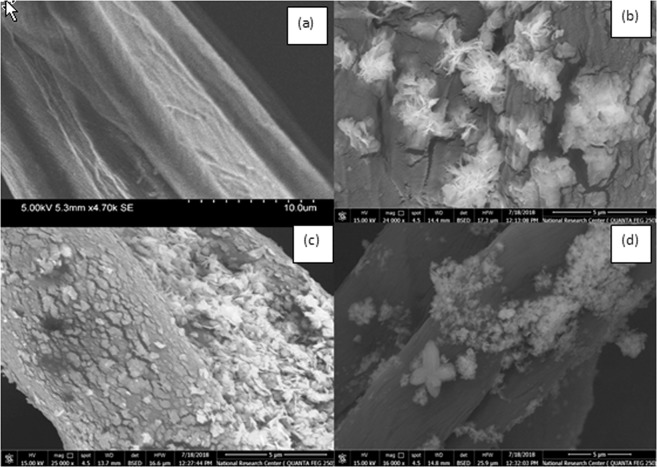
SEM images of (**a**) blank cotton (quoted from our previous work (ref. ^3^)) (**b**) ZnO/cotton (**c**) ZnO-Ag/cotton (**d**) ZnO/starched cotton.

The morphology of blank cotton, ZnO/cotton, ZnO-Ag/cotton and ZnO/starched cotton (3% wt. starch) is examined by SEM and is presented in Fig. 5 (images a–d)^3^. The SEM image for the blank cotton fiber (Fig. 5, image a) shows grooves and fibrils on the surface of the fiber. SEM image of ZnO/cotton (Fig. 5, image b) shows that ZnO-NPs were agglomerated and fully covered all cotton surface fibers with nano shells of ZnO with no defined boundaries appeared onto the fiber surface. The agglomerates of ZnO-nanoparticles were dense and compact; some ZnO-NPs appears as sea stars. In the case of SEM image of ZnO-Ag/cotton (Fig. 5, image c), the ZnO-Ag nanocomposite appears as dense smaller flakes of well adhered to the cotton fiber. Some particles are seen as flakes of different sizes or interconnected nano-sheets, others were seen as nano-flowers (Fig. 5, image c). SEM image of ZnO/cotton-starch (Fig. 5, image d), the surface of the fabric was covered with interconnected ZnO-NPs, some are seen as nano flowers (Fig. 5, image d).

#### X-Ray Diffraction (XRD)

**Figure 6:**
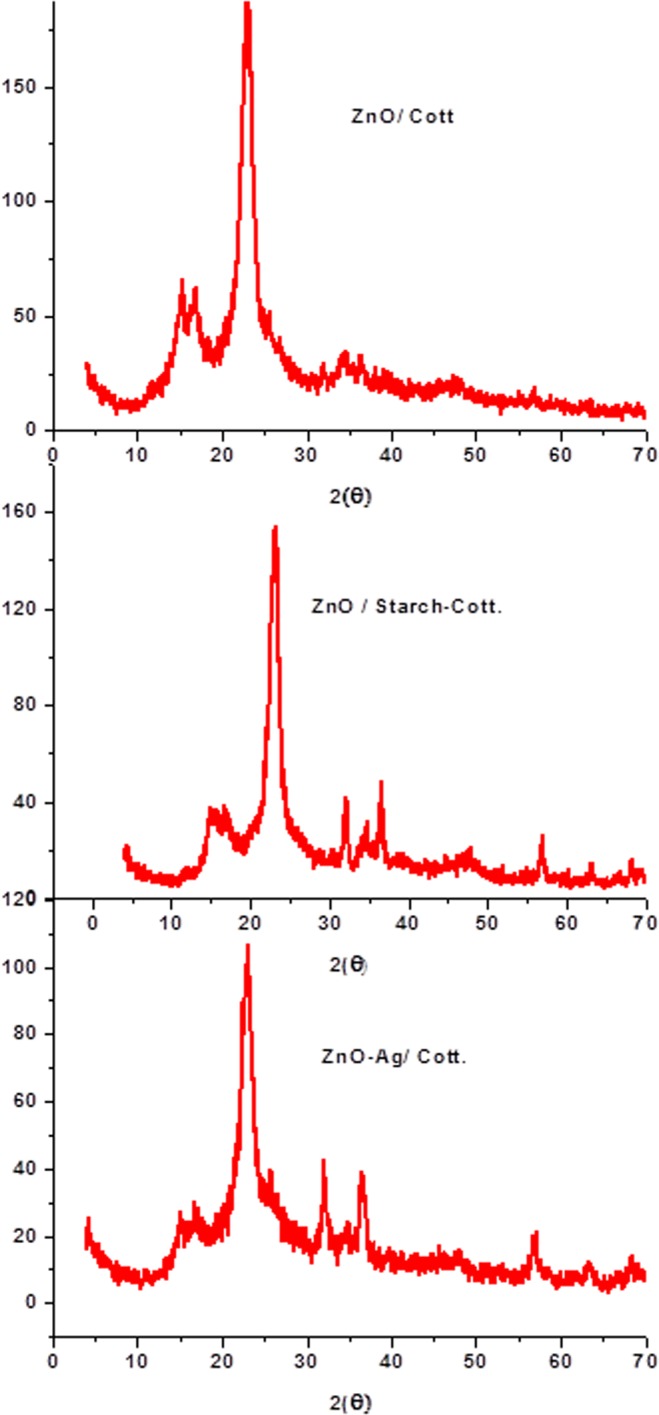
XRD analysis of ZnO/cotton, ZnO/starched-cotton and ZnO-Ag/cotton.

The XRD patterns for ZnO/cotton and ZnO/starched cotton and ZnO-Ag/cotton are presented in Fig. 6. The diffraction pattern of ZnO-NPs was well indexed to the hexagonal ZnO wurtzite structure^3,9,26^ In the case of ZnO-Ag composite there was no growth along 002 plane, this probably due to the interaction with Ag-NPs along the 002 plane. The main three peaks at 2θ = 31.5, 35 and 37 can be indexed to the lattice planes at 100, 002 and 101. These values were closely similar to the previously reported values^3^. The diffraction peaks at diffraction angle below 30 are characteristic with cellulosic cotton material^3^. Diffraction peaks due to the presence of impurities were not observed in the XRD patterns, confirming a high purity of the synthesized composites. The mean crystallite particle size of the ZnO (11.5 nm) in the ZnO-Ag/cotton calculated by Scherrer’s equation is smaller than the sized of ZnO in ZnO/cotton or ZnO/starched cotton (16.2 nm). This is probably due to interaction with silver nanoparticles, which may reduce the size of ZnO particles. This is was also evident from TEM analysis. In Fig. 6 the reverse is present: the intensity of XRD peaks for ZnO/starch-cotton is much higher than that of ZnO/cotton. The reason for this change is that higher content of ZnO particles were loaded onto the starched cotton than un-starched cotton (see Fig. 4).

#### TEM analysis

**Figure 7:**
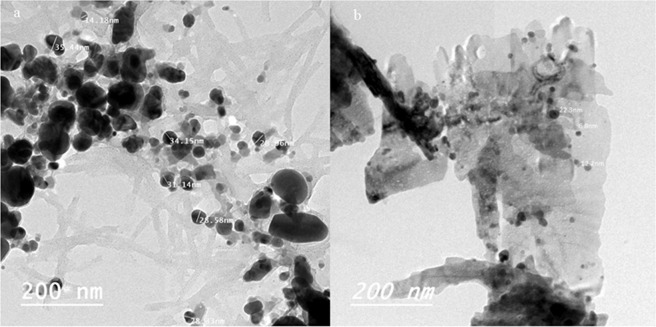
TEM image of (**a**) Ag-NPs, CuO-Ag and (**b**) ZnO-Ag/cotton nanocomposite.

TEM image of the Ag-NPs sol (Fig. 7a) reveals that there were some agglomerations of Ag-NPs result of formation of large sphere or rod like particles in the range 20–50 nm, similar results are also recently reported. TEM analysis of ZnO-Ag nanocomposite coated cotton is displyed in Fig. 7b. The TEM-image showed that the ZnO-Ag (black color) have smaller particle size of 10–30 nm than that of Ag-NPs.

#### Antimicrobial tests

**Figure 8:**
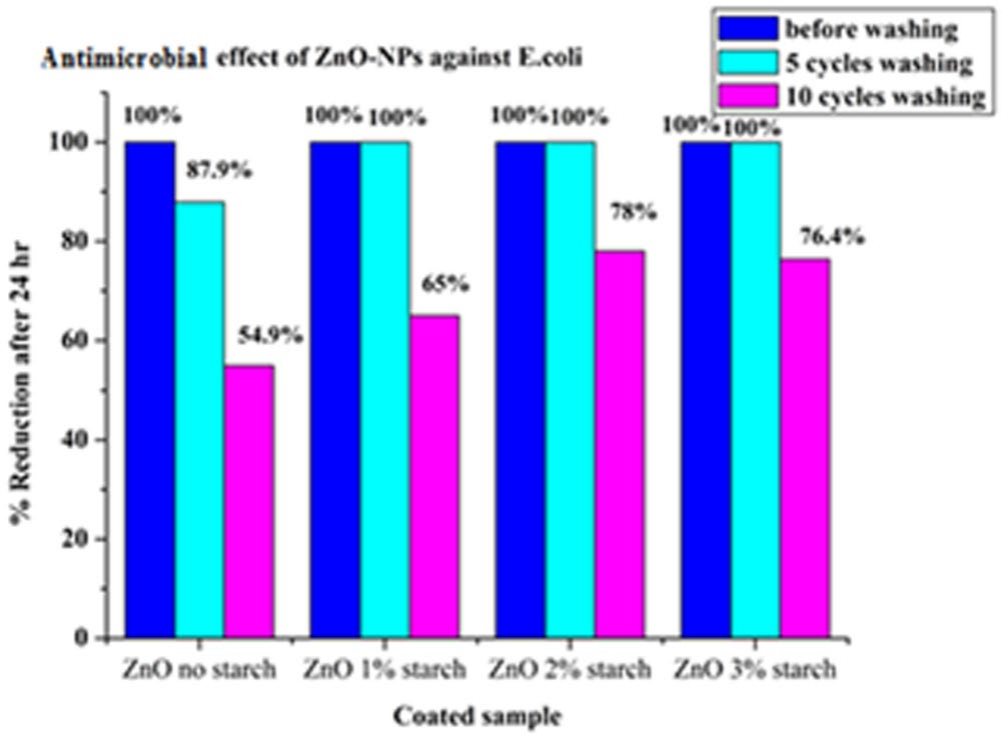
Antimicrobial effect of ZnO/cotton and ZnO/starched cotton (1–3%).

**Figure 9:**
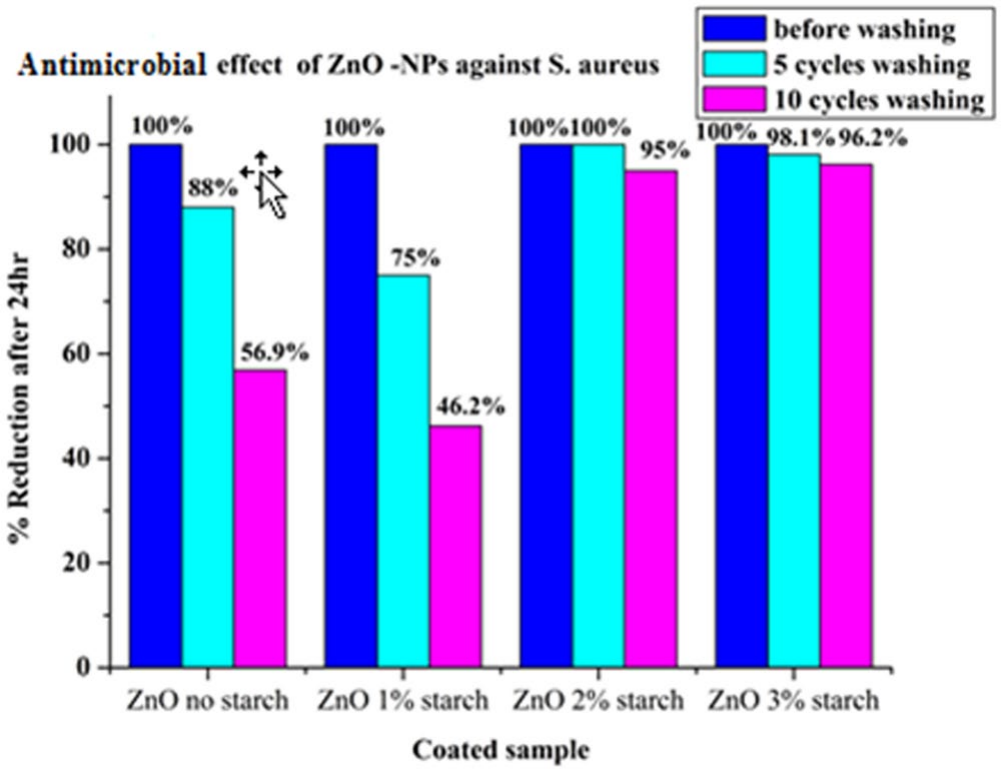
Antimicrobial effect of ZnO/cotton and starched/cotton (1–3%) against aureus.

The antibacterial activity of the ZnO/cotton and ZnO/cotton-starch were applied according to the^3,28,34^ standard method AATCC 100. The reduction percentage (%) was determined by the following formula R = 100(B − A)/B, where R = reduction percentage (%), A = the number of microbial cells recovered from the inoculated coated cotton material incubated 24 hrs contact period, B = the number of microbial cells recovered from the inoculated uncoated cotton material immediately after inoculation (at “0” contact time). The results were compared with that of the control samples and with that of the uncoated fabrics sample. The antimicrobial activity against *E. coli* and *S*. aureus for ZnO-NPs coated cotton and coated starched cotton are given in Figs. 8 and 9. The antimicrobial results of the ZnO-NPs coated starched cotton have better antimicrobial activity against *E. coli* and *S*. aureus than the corresponding ZnO-NPs coated cotton with no starch. The reason for this behavior is that more content of ZnO-NPs is loaded onto the cotton fabric in presence of starch, the antimicrobial activity increases with increasing the starch percentage (%). The antimicrobial results shown the highest bacterial reduction where found when used 3% starch after washed 5 cycles and 10 cycles by compared to 2% or 1% starch against two types of bacteria.

The antimicrobial activities of ZnO-NPs coated cotton or coated starched cotton against *E. coli* and *S*. aureus after 10 cycles test in “antibacterial activity test” were compared with some previous studies (Table 1). The data support the excellent activity of using starched cotton as stabilizing for ZnO.

**Table 1:** Reduction of antimicrobial activity of ZnO-NPs coated cotton or coated starched cotton against *E. coli* and *S*. aureus after 10 cycles.

Material	Reduction of *E. coli* (%)	Reduction of S. aureus (%)	Ref.
ZnO/cotton	54	57	This work
ZnO/cotton-starch(3%)	76	96	This work
ZnO/cotton-SDS	89	91	ref. ^3^
ZnO/cotton-HY	90	92	ref. ^3^
ZnO/cotton-enzyme	98	30	ref. ^28^
ZnO/cotton–enzyme	50	1.0	ref. ^28^

**Figure 10:**
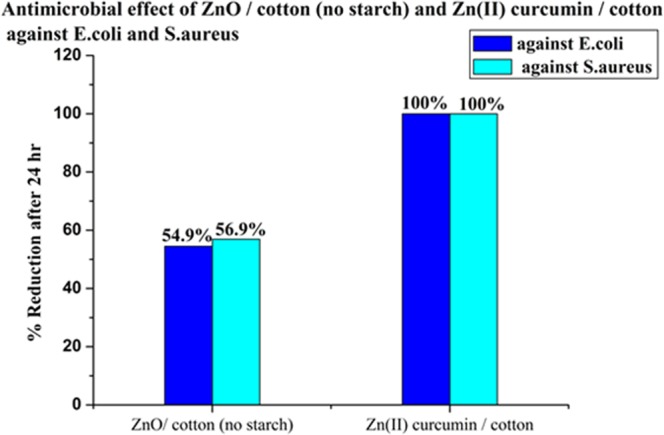
Antimicrobial of ZnO/cotton and Zn(II) curcumin against *E. coli* & S. aureus.

The antibacterial activity of the ZnO/cotton and Zn(II) curcumin/cotton for *E. coli* and S. aureus are given in Fig. 10. The Zn(II) curcumin complex showed significant higher reduction of 100% for both *E. coli* and S. aureus when compared with that of the ZnO/cotton (Fig. 10). The reason for this behavior is that Zn(II) curcumin complex showed a better antimicrobial better than that of ZnO-NPs or free cucumin^33^.

**Figure 11:**
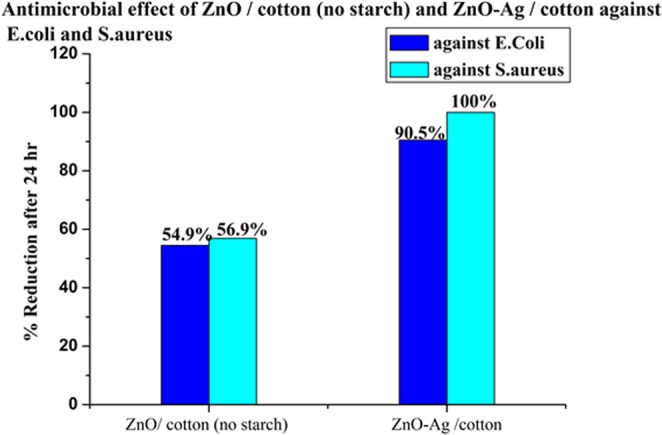
Antimicrobial of ZnO/cotton and ZnO-Ag/cotton materials against *E. coli* & S. aureus.

The antimicrobial activity for ZnO-Ag/cotton for *E. coli* and S. aureus is higher than that of ZnO/cotton (Fig. 11). The ZnO-Ag nanocomposite showed highest reduction of both *E. coli* and S. aureus than that of ZnO-NPs. This is probably that the synergistic antibacterial activity of ZnO-Ag nanohybrid material on Gram-positive and Gram-negative bacteria is found to be more effective, compared to the individual component ZnO or Ag)^35,36^. This is was evident from Fig. 11. This can be explained that ZnO-Ag has smaller size than ZnO-NPs, so it shows more effective reduction for both types of bacteria (TEM).

## Conclusion

Zinc oxide nanoparticles were prepared and deposited onto cotton and starched cotton fabrics. The starched cotton was selected over pristine cotton as substrate, because starch (corn) is none toxic and compatible with cotton material substrate. Starch acts as modifying agent to improve the adhesion properties of the cotton for better deposition of zinc oxide nanoparticles. This apparently decreases the leaching the ZnO-NPs from the cotton fabric surface and increased remarkably its antimicrobial activity. ZnO-NPs coated cotton materials were functionalized with silver nanoparticles and curcumin, ZnO-Ag nanohybride and Zn(II) curcumin complex coated cotton were formed, respectively. ZnO-Ag/cotton material illustrated synergistic and enhanced antimicrobial potency for both gram-positive and gram- negative bacteria attributed to the strong interaction between semiconductor ZnO and metallic Ag. It is found that the antimicrobial activity results for Zn(II) curcumin complex/cotton is significantly enhanced when the curcumin solution is added to the ZnO/cotton forming Zn(II) complex.

## References

[1] Perelshtein I, Lipovsky I, Perkas N, Tzanov T, Gedanken A (2016). Sonochemical co-deposition of antibacterial nanoparticles and dyes on textiles. Beilstein J. Nanotechnol..

[2] Salat M (2018). Durable antimi-crobial cotton textiles coated sonochemically with ZnO nanoparticles embedded in an *in-situ* enzymatically generated bioadhesive. Carbohydr. Polym..

[3] El-Nahhal MI (2017). Stabilization of nano-structured ZnO particles onto the surface of cotton fibers using different surfactants and their antimicrobial activity. Ultrason. Sonochem..

[4] Borah JP, Barman J, Sarma KC (2008). Structural and optical properties of ZnS nanoparticles. Chalcogenide Lett..

[5] Wang H (2011). ZnO films grown on cotton fibers surface at low temperature by simple two-step process. Mater. Lett..

[6] Borkow G, Gabbay J (2009). Copper, an ancient remedy returning to fight microbial and viral infections. Curr. Chem. Biol..

[7] Abramov OV (2009). Pilot scale sonochemical coating of nanoparticles onto textile to produce biocidal fabrics. Surf Coat Technol..

[8] El-Nahhal IM (2013). Nano-structured zinc oxide–cotton fibers: synthesis, characterization and applications. J. Mater. Sci.: Mater..

[9] El-Nahhal IM (2012). Nanostructured copper oxide-cotton fibers: synthesis, characterization, and applications. Int. Nano Lett..

[10] Perelshtein I (2009). CuO-cotton nanoparticles: formation, morphology and antibacterial activity. Surf Coat Technol..

[11] Xu Q (2018). Preparation of copper nanoparticles coated cotton fabrics with durable antibacterial properties. Fibers and Polymers.

[12] Xu Q, Wu Y, Zhang Y, Fu F, Liu X (2016). Durable antibacterial cotton modified by silver nanoparticles and chitosan derivative binder. Fibers and Polymers.

[13] Li Z (2017). The room temperature electron reduction for the preparation of silver nanoparticles on cotton with high antimicrobial activity. Carbohydrate Polymers.

[14] Xu Q (2019). One-pot fabrication of durable antibacterial cotton fabric coated with silver nanoparticles via carboxymethyl chitosan as a binder and stabilizer. Carbohydrate Polymers.

[15] Makhluf S (2005). Microwave-Assisted Synthesis of Nanocrystalline MgO and Its Use as a Bacteriocide. Adv. Funct. Mater..

[16] Manna Adhar C. (2011). Synthesis, Characterization, and Antimicrobial Activity of Zinc Oxide Nanoparticles. Nano-Antimicrobials.

[17] Singh G, Joyce EM, Beddow J, Mason TJ (2012). Evaluation of antibacterial activity of ZnO nanoparticles coated sonochemically onto textile fabrics. J. Microbiol. Biotechnol. Food Sci..

[18] Firdhouse MJ, Lalitha P (2013). Fabrication of Antimicrobial Perspiration Pads and Cotton Cloth Using Amaranthus dubius Mediated Silver Nanoparticles. J. Chem..

[19] Galkina OL (2004). The sol–gel synthesis of cotton/TiO2 composites and their antibacterial properties. Surf Coat Technol..

[20] Khosravian S, Montazer M, Malek RM, Harifi T (2015). *In situ* synthesis of nano ZnO on starch sized cotton introducing nano photo active fabric optimized with response surface methodology. Carbohydr. Polym..

[21] Hong KH (2014). Preparation and properties of multifunctional cotton fabrics treated by phenolic acids. Cellulose..

[22] Zhang D, Chen L, Zang C, Chen Y, Lin H (2013). Antibacterial cotton fabric grafted with silver nanoparticles and its excellent laundering durability. Carbohydr. Polym..

[23] Dhiman G, Chakraborty JN (2015). Antimicrobial performance of cotton finished with triclosan, silver and chitosan. Fashion and Textiles..

[24] El Shafei A, Abou-Okeil A (2011). ZnO/carboxymethyl chitosan bionano-composite to impart antibacterial and UV protection for cotton fabric. Carbohydr. Polym..

[25] Rosca C, Popa MI, Lisa G, Chitanu GC (2005). Interaction of chitosan with natural or synthetic anionic polyelectrolytes. 1. The chitosan–carboxymethylcellulose complex, *Carbohydr*. Polym..

[26] Varaprasad K, Raghavendra GM, Jayaramudu T, Seo J (2016). Nano zinc oxide– sodium alginate antibacterial cellulose fibres. Carbohydr. Polym..

[27] Ghayempour S, Montazer M (2017). Ultrasound irradiation based *in-situ* synthesis of star-like Tragacanthgum/zinc oxide nanoparticles on cotton fabric. Ultrason. Sonochem..

[28] Petkova P, Francesko A, Perelshtein I, Gedanken A, Tzanov T (2016). Simultaneous sono-chemical-enzymatic coating of medical textiles with antibacterial ZnO nanoparticles. Ultrason. Sonochem..

[29] El-Nahhal MI (2018). The efficacy of surfactants in stabilizing coating of nano-structured CuO particles onto the surface of cotton fibers and their antimicrobial activity. Mater. Chem. Phys..

[30] Macedo J, Daniel B, Rocha, Derval SR (2017). Green recoating of cotton fiber by different starching methods. *J*. Materials: Design and Applications.

[31] Joydeb M, Srishti G, Nagaraju S, Nivedita S, Rohit KR (2015). Biomimetic Method To Assemble Nanostructured Ag@ZnO on Cotton Fabrics: Application as Self-Cleaning Flexible Materials with Visible-Light Photocatalysis and Antibacterial Activities. ACS Appl. Mater. Interfaces..

[32] Sofia M, Costa DP, Ferreira AF, Filipe V, Fangueiro R (2018). Multifunctional Flax Fibres Based on the Combined Effect of Silver and Zinc Oxide (Ag/ZnO) Nanostructures. Nanomaterials..

[33] Hatamie S (2012). Complexes of cobalt nanoparticles and polyfunctional curcumin as antimicrobial agents. Mater. Sci. Eng. C..

[34] Vary FuG, Lin PSC (2005). Anatase TiO2 nanocomposites for antimicrobial coatings. J. Phys. Chem. B..

[35] Ghosh S (2012). ZnO/Ag nanohybrid: Synthesis, characterization, synergistic antibacterial activity and its mechanism. RSC ADVANCES.

[36] Zare M (2019). Novel Green Biomimetic Approach for Synthesis of ZnO-Ag Nanocomposite; Antimicrobial Activity against Food-borne Pathogen, Biocompatibility and Solar Photocatalysis. Scientific Reports.

