# Author Correction: Preparation and antimicrobial activity of ZnO-NPs coated cotton/starch and their functionalized ZnO-Ag/cotton and Zn(II) curcumin/cotton materials

**DOI:** 10.1038/s41598-020-65025-w

**Published:** 2020-05-27

**Authors:** Issa M. El-Nahhal, Jamil Salem, Rawan Anbar, Fawzi S. Kodeh, Abdelraouf Elmanama

**Affiliations:** 1 Department of Chemistry, Al-Azhar University, P O Box 1277, Gaza, Palestine; 2 Department of Medical laboratory sciences, Islamic University of Gaza, P.O Box 108, Gaza, Palestine

Correction to: *Scientific Reports* 10.1038/s41598-020-61306-6, published online 25 March 2020

The original version of this Article contained a typographical error in the spelling of the author Abdelraouf Elmanama, which was incorrectly given as Abedelraouf Elmanama. This has now been corrected in the PDF and HTML versions of the Article.

Additionally, the Author Contributions section has been corrected.

“Issa El Nahhal wrote the main manuscript, Rawan Anbar conduct the experimental part and determined all durability tests, Jamil Salem and Fawzi conduct the XRD, SEM, TEM and UV-vis analysis. Abedelraouf Elmanama has conducted all antimicrobial tests.”

now reads:

“Issa El Nahhal wrote the main manuscript, Rawan Anbar conduct the experimental part and determined all durability tests, Jamil Salem and Fawzi conduct the XRD, SEM, TEM and UV-vis analysis. Abdelraouf Elmanama has conducted all antimicrobial tests.”

